# Heparan sulfates and heparan sulfate binding proteins in sepsis

**DOI:** 10.3389/fmolb.2023.1146685

**Published:** 2023-02-14

**Authors:** Yi-En Liao, Jian Liu, Katelyn Arnold

**Affiliations:** Division of Chemical Biology and Medicinal Chemistry, Eshelman School of Pharmacy, University of North Carolina, Chapel Hill, NC, United States

**Keywords:** heparan sulfate, heparin, heparan sulfate binding proteins, HMGB1 (high mobility group box 1), histones

## Abstract

Heparan sulfates (HSs) are the main components in the glycocalyx which covers endothelial cells and modulates vascular homeostasis through interactions with multiple Heparan sulfate binding proteins (HSBPs). During sepsis, heparanase increases and induces HS shedding. The process causes glycocalyx degradation, exacerbating inflammation and coagulation in sepsis. The circulating heparan sulfate fragments may serve as a host defense system by neutralizing dysregulated Heparan sulfate binding proteins or pro-inflammatory molecules in certain circumstances. Understanding heparan sulfates and heparan sulfate binding proteins in health and sepsis is critical to decipher the dysregulated host response in sepsis and advance drug development. In this review, we will overview the current understanding of HS in glycocalyx under septic condition and the dysfunctional heparan sulfate binding proteins as potential drug targets, particularly, high mobility group box 1 (HMGB1) and histones. Moreover, several drug candidates based on heparan sulfates or related to heparan sulfates, such as heparanase inhibitors or heparin-binding protein (HBP), will be discussed regarding their recent advances. By applying chemical or chemoenzymatic approaches, the structure-function relationship between heparan sulfates and heparan sulfate binding proteins is recently revealed with structurally defined heparan sulfates. Such homogenous heparan sulfates may further facilitate the investigation of the role of heparan sulfates in sepsis and the development of carbohydrate-based therapy.

## Introduction

Sepsis, a lethal syndrome caused by dysregulated host responses to infection, leads to millions of deaths annually (Singer et al., 2016). Several underlying mechanisms of sepsis have been identified, including overwhelming inflammation and immune suppression, resulting in cell damage and organ dysfunction. Heparan sulfates (HSs) are enriched in the protective cell surface glycocalyx layer and are shed in septic patients. Recent research identifies pro-inflammatory molecules that bind to HSs, including high mobility group box 1 (HMGB1) and extracellular histones. In addition, HSs are found to interact with bacterial-associated molecules, such as the lipopolysaccharide (LPS)/HMGB1 complex and the heparin-binding protein (HBP).

**FIGURE 1:**
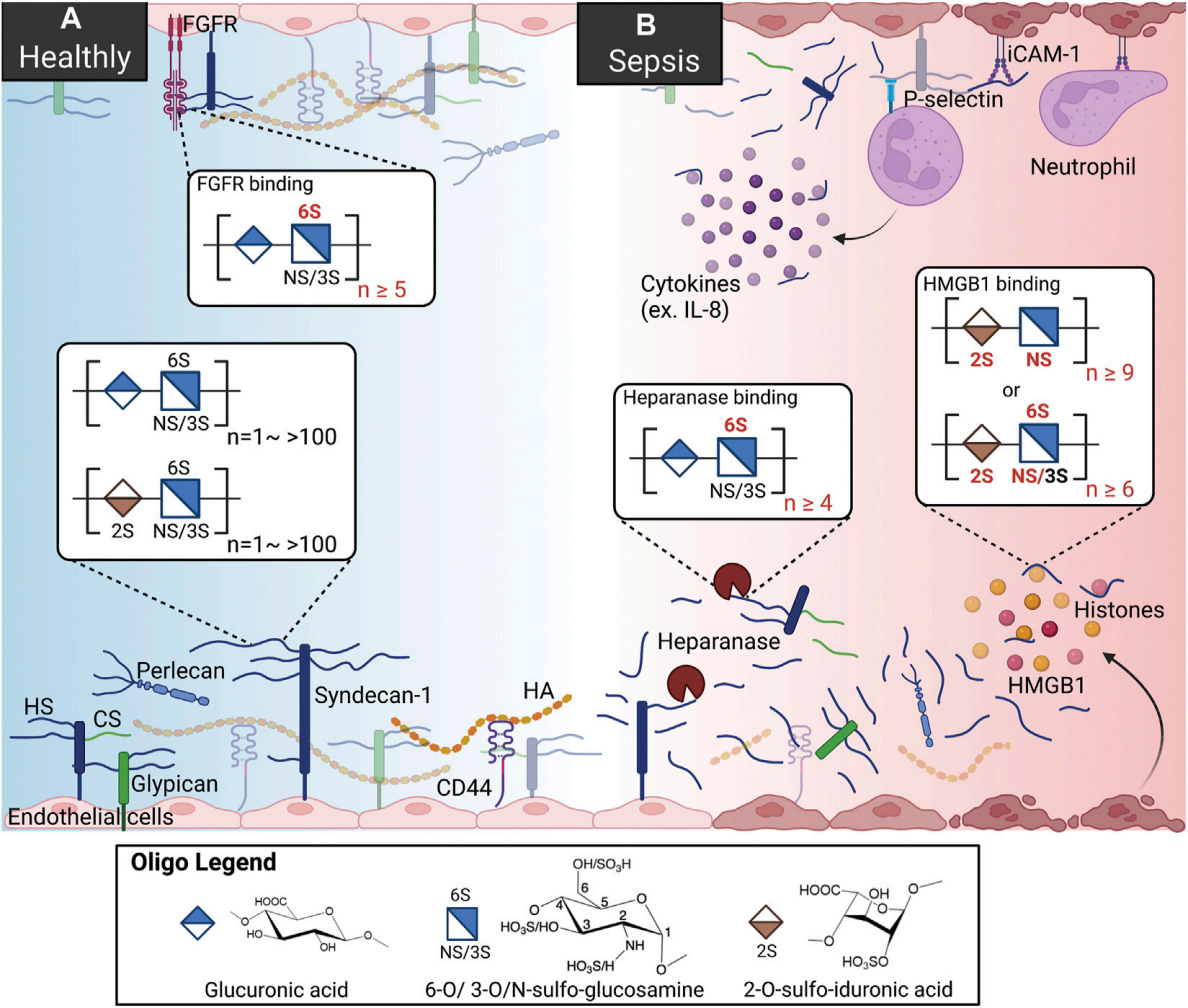
HSs and HSBPs on glycocalyx in health and sepsis. **(A)** Heparan sulfates (HSs) present on the cell surfaces as proteoglycans, binding to perlecan, syndecans-1, and glypican. With other glycoproteins, including chondroitin sulfate (Tam et al., 2008) and hyaluronan (HA) bound to syndecans-1 and CD44, HS proteoglycans form the glycocalyx and regulate cell signaling by interacting with HS binding proteins (HSBPs) such as fibroblast growth factor receptor (FGFR), intracellular adhesion molecule-1 (iCAM-1). Binding to HSBPs requires specific HS structures; for example, FGFR binding requires 6-O-sulfated glucosamine and more than ten saccharide units. **(B)** During sepsis, HSs are released by upregulated heparanase. HSs mediate inflammation by binding to dysregulated HSBPs, such as extracellular histones, high mobility group box 1 (HMGB1), and interleukin 8 (IL-8). HSs also serve as co-receptors for immune cells by binding to p-selectin. Specific sulfation and length are also required for HS binding to heparanase and HMGB1.

HSs are linear polysaccharides made in the Golgi compartment by various biosynthetic enzymes, consisting of repeating disaccharides of glucuronic acid (GlcA) and glucosamine (GlcN) or iduronic acid (IdoA). HSs can include two to over a hundred disaccharide units, depending on the polymerization process catalyzed by the exostosin glycosyltransferases 1/2 (EXT 1/2). The structures of HSs are diversified by sulfation patterns. Different sulfotransferases and epimerization of glucuronic acid to iduronic acid are involved in HS chain modification by N-deacetylase/N-sulfotransferase (NDST), C_5_ epimerase, and 2-/3-/6-O-sulfotransferases (2-/3-/6-OST) (Sarrazin et al., 2011). HSs are usually secreted by exocytosis and present on the cell surfaces alone or attached to proteoglycans (Figure 1).

Collectively with other glycoproteins, glycosaminoglycans (GAG), and plasma proteins, proteoglycans make up the glycocalyx that carpets the luminal side of endothelial cells lining every blood vessel in the vascular system (Zeng, 2017).

The gel-like layer of the glycocalyx is essential for normal physiology in several aspects: it regulates the permeability of the vasculature, serves as a mechanotransducer, and functions as an anti-inflammatory and anticoagulant coat to protect the underlying endothelium. The main components of the glycocalyx are membrane-bound proteoglycans, i.e., syndecans and glypicans, and soluble proteoglycan, i.e., perlecan. In the vasculature, HS proteoglycans represent over half the total amount of proteoglycans present in the glycocalyx (Ihrcke et al., 1993). Multiple functions of the glycocalyx are carried out by the interactions of HSs and hundreds of HS binding proteins (HSBPs), which cover a wide range of biological functions, such as mediating signaling pathways, maintaining glycocalyx integrity, and vascular homeostasis (Xu et al., 2018). For example, HSs regulate the activation of fibroblast growth factor 2 (FGF2) -FGF receptor 1 (FGFR1) signaling for the reconstitution of glycocalyx (Figure 1A) (Yang et al., 2017). The detailed functions of the glycocalyx can be found in other reviews (Ihrcke et al., 1993; Oshima et al., 2021).

It is well known that in pathologic conditions like sepsis, atherosclerosis, and kidney disease, the glycocalyx is damaged, resulting in vascular hyperpermeability and increased apoptosis (Figure 1B) (Garsen et al., 2016; Puerta-Guardo et al., 2016; Uchimido et al., 2019). Heparanase is an endo-β-glucuronidase that cleaves HSs. The expression and activity of heparanase are frequently associated with glycocalyx damage. Loss of glycocalyx HSs leads to the deterioration of glycocalyx function. The process is described as HSs shedding, which releases HSs in various sizes and sulfation patterns into circulation. HSs are responsible for multiple biological functions through interaction with various proteins in a homeostatic state; therefore HSs shedding interrupts crosstalk and communications between cells, contributing to abnormal inflammation, coagulation, and lipid metabolism systems under septic conditions (Hayashida et al., 2009; Arnold et al., 2020a). In an LPS-induced sepsis mouse model, pulmonary endothelial heparanase was activated in a TNF-α dependent manner (Schmidt et al., 2012). Consequently, the increase in heparanase activity leads to the development of acute lung injury during endotoxemia. In his study, the authors also demonstrate a high heparanase expression in biopsies of human lung tissue with diffuse alveolar damage, suggesting the potential contribution of heparanase to lung injury. Moreover, glycocalyx reconstitution is impaired in sepsis. A report showed that although the HS fragments are able to activate FGF2-FGFR1 signaling, HS biosynthesis by EXT1 and FGFR1 expression are downregulated in sepsis (Yang et al., 2017). Since the damaged glycocalyx cannot be repaired in sepsis, the loss of vascular homeostasis may contribute to long-term organ injury and dysfunction. This review focuses on the role of HSs and several dysregulated proteins in sepsis and therapeutic strategies reported preclinically and clinically.

## The HSBPs HMGB1 and extracellular histones in sepsis

HSs have been found to interact with various dysregulated proteins in preclinical and clinical trials, supporting its critical role in sepsis pathology. In this review, we will focus on two critical HSBPs, HMGB1 and extracellular histones. Other HSBPs that were studied in sepsis animal models or clinical trials are summarized in Table 1.

**TABLE 1:** Dysregulated HSBPs in sepsis and the structural selectivity from HSs.

Proteins	Function in sepsis	HS binding effects and preference	References
Cytokines/chemokines			
IL-8	• Recruit and activate neutrophils and other immune cells	• Endothelial HSs: promote IL-8 dimerization and neutrophils transmigration	Axelsson et al. (2012), Goodall et al. (2014), Jayson et al. (2015), Joseph et al. (2015)
	• Correlate to sepsis-induced organ injury, especially lungs	• Heparin: reduce LPS induced IL-8	
		• Heparin derivatives: IL-8 binding affinity is length dependent (dp14 > dp8 > dp4 > dp2); IL-8 dimer requires ≥dp8, monomer ≥dp4	
		• HS 12-mer: 6-O-sulfation at the reducing end is required to inhibit IL-8	
MIP-2 (CXCL2)	• Recruit neutrophils and macrophages	• Endothelial HSs: mediate MIP-2 gradients	Hayashida et al. (2009), V Kumar et al. (2015)
	• Correlated to acute lung injury	• Syndecan-1/HSs: facilitate MIP-2 removal	
KC (CXCL1)	• Essential for neutrophil migration, neutrophil-related bacterial clearance, NET production	• Cell surface HSs: stabilize CXCL1/CXCR2 binding, can be blocked by heparin	Wang et al. (2003), Hayashida et al. (2009), Poluri et al. (2013), Jin et al. (2014)
	• Modulate T cell function *via* IL-17A	• HS 8-mer: bind and stabilize CXCL1 dimer	
		• Syndecan-1/HSs: facilitate KC removal	
MCP-1 (CCL2)	• Recruit monocytes	• HS 8-mer: prevent MCP-1 dimer dissociation; induce tetramer formation	Lau et al. (2004), Gomes et al. (2013), Seo et al. (2013), Goodall et al. (2014), Matsumoto et al. (2018)
	• Mediate bacterial clearance	• Heparin: reduce LPS induced MCP-1	
	• Elevate in the acute phase of sepsis, correlate to sepsis severity	• HS sulfation is required but independent of sulfation sites	
MIP-1β (CCL4)	• Potential survival predictor in septic shock	• Heparin: bind to MIP-1β; the binding may be important for MIP-1β activity	Koopmann et al. (1999), Proudfoot et al. (2003), Nowak et al. (2010)
RANTES (CCL5)	• Released from platelets or T cells to recruit neutrophils	• Heparin: binds to RANTES; the binding is essential for RANTES activity; RANTES/CCR1 can be blocked by HSs/heparins	Hwaiz et al. (2015), Singh et al. (2015), Liang et al. (2016)
	• Contribute to septic acute lung injury	• HS 4-mer: HS sulfation dependent but independent of sites	
		• HS: 2S6SNS 2-mer/4-mer/6-mer/8-mer tested, 8-mer stabilize RANTES	
Adhesion molecules			
L-selectin	• Mediate leukocytes migration and adhesion to endothelium	• Endothelial HSs: essential for L-selectin mediated neutrophil trafficking	Seidelin et al. (2002), Wang et al. (2002), Wang et al. (2005)
	• Soluble L-selectin is lower in septic patients	• Heparin: binds and inhibits L-selectin associated inflammation	
		• HS 6-O-sulfation dependent	
P-selectin	• Mediate leukocyte recruitment and adhesion at the infected site	• Heparin: binds and inhibits P-selectin binding to endothelial cells and associated inflammation	Luo et al. (2001), Wang et al. (2002), De Stoppelaar et al. (2015)
	• Upregulated in endothelial cells and platelets during sepsis	• HS 6-O-sulfation dependent	
iCAM-1	• Upregulated in endothelial cells to mediate leukocyte adhesion in sepsis	• Heparin: reduce LPS induced iCAM-1	Luan et al. (2014), Woodfin et al. (2016), Ode et al. (2018), Olivares-Silva et al. (2018)
	• Upregulated in neutrophils in sepsis, correlated to increasing NET, phagocytosis, ROS generation	• LMWH: reduce iCAM-1 expression in lung of endotoxemia mice	
		• HSs from bovine kidney: increase iCAM-1 expression in cardiac fibroblast	
Lipid Metabolism			
ApoB/LDL	• ApoB presents in LDL and VLDL. LDL/VLDL enhances the LPS-binding capacity of LBP	• Heparin: bind to LDL by interacting with apoB; trigger VLDL remodeling	Vreugdenhil et al. (2001), Deng et al. (2012), Felici et al. (2021), Jayaraman et al. (2022)
	• LDL reduces in septic patients	• Syndecan-1: HS shedding in sepsis may impair VLDL clearance, cause hypertriglyceridemia	
ApoE	• ApoE presents in HDL, LDLR	• HS proteoglycans: binds to apoE through sulfated HSs, causing apoE-containing lipoprotein localization and clearance	Libeu et al. (2001), Van Oosten et al. (2001), Kattan et al. (2008), Chuang et al. (2011)
	• ApoE administration protects against LPS-induced mortality but worsens CLP-induced mortality in mice	• Heparin: protect against CLP-induced sepsis, may be *via* inhibiting apoE-LDLR binding and uptake	
Coagulation			
AT III	• Anticoagulant with potential anti-inflammatory effects, binding to factor Xa, factor IIa, syndecans-4	• Heparin: binds to AT III, amplifying its binding to factor Xa/IIa; co-treatment with AT III increases the risk of hemorrhage	Warren et al. (2001), Iba et al. (2012b), Ishikawa et al. (2017), Arnold et al. (2020a), Schlömmer et al. (2021)
	• Protects against LPS-induced endotoxemia		
	• May have clinical benefits for septic patients with DIC	• HS binding sequence identified as GlcNS/Ac6S-GlcA-GlcNS3S ± 6S-IdoA2S-GlcNS6S	
	• Anticoagulant by inhibiting tissue factor-factor VIIa complex	• Heparin: binds to TFPI, releasing it and amplifying its binding to factor FVIIa; co-treatment with TFPI does not consistently reduce the mortality rate in septic patients	Valentin et al. (1994), Xu et al. (2002), Abraham et al. (2003), Tang et al. (2007), Semeraro et al. (2012)
TFPI	• Decrease expressions in endothelial cells in septic baboon	• Heparin derivatives: 2-mer to 18-mer tested, binding affinity is length dependent; 14-mer inhibitory effect close to UFH	
	• May have clinical benefits for septic patients		
Others			
HBP (Azurocidin)	• Released from neutrophil, increasing endothelial permeability and neutrophil migration	• Heparin/LMWH: block HBP induced lung injury, mitochondrial dysfunction, and IL-6 release, maybe through competition with endothelial HS binding	Rao et al. (2010), Wang et al. (2014b), Bentzer et al. (2016), Fisher and Linder (2017), Fisher et al. (2017)
	• Potential biomarker for sepsis-induced organ dysfunction		
LBP	• Increase in septic patients, mediate acute kidney injury	• Heparin derivatives: sulfation dependent; N-/O-desulfation eliminate binding ability	Opal et al. (1999), Heinzelmann and Bosshart (2005), Stasi et al. (2017), Simpson and Trent (2019)
	• Bind and present LPS to CD14 and TLR4 on cell surfaces, enhance LPS recognition, or neutralize LPS	• Heparin/LMWH: bind to LPS and transfer LPS to CD14, but not fondaparinux	
Heparanase	• HS specific endoglycosidase		Peterson and Liu (2012), Lygizos et al. (2013), Matan et al. (2018), Uchimido et al. (2019)
	• Increase and activate in CLP/LPS induced sepsis, degrade glycocalyx and contribute to acute lung injury	• HSs: cleavage of the linkage of glucuronic acid linked to 6-O-sulfated glucosamine	
	• Reduce in severe septic patients	• Heparin/HSs: heparanase inhibitor, protect against glycocalyx degradation	
Kallistatin	• Plasma kallikrein inhibitor with anti-angiogenic and anti-inflammatory effect in septic mice	• Heparin: binding site of heparin identified, essential for kallistatin anti-inflammatory effect targeting HMGB1/NF-κB	Chen et al. (2001), Li et al. (2014a), Lin et al. (2015), Lin et al. (2017)
	• Decrease in septic patients and correlated with sepsis severity		

*IL-8, interleukin-8; MIP-2/1β, macrophage inflammatory protein 2/1β; CXCL1/2, chemokine (C-X-C motif) ligand 1/2; KC, keratinocyte chemoattractant; MCP-1, monocyte chemoattractant protein 1; CCL1/2/4/5, chemokine (C-C motif) ligand 1/2/4/5; RANTES, regulated upon activation, normal T cell expressed and presumably Secreted; iCAM-1, intercellular adhesion molecule 1; ApoB/E, apolipoprotein B/E; LDL, low-density lipoprotein; ATIII, antithrombin III; TFPI, tissue factor pathway inhibitor; HBP, heparin-binding protein; LBP, LPS, binding protein. *dp2 = 2-mer = 2 saccharide units, dp18 = 18-mer = 18 saccharide units.

Although various HSBPs have been identified, many structure-function relationships between HSs and the proteins in sepsis remain unclear. As shown in Table 1, most studies were conducted with heparin, an HS mixture with anticoagulant activity. The structural complexity of heparin makes it challenging to elucidate the effect of size and sulfation patterns on specific protein binding. Furthermore, the dominating anticoagulant activity of heparin makes it impossible to take therapeutic advantage of its chemokine/cytokine binding, inhibition of adhesion molecules, and effects on lipid metabolism that heparin is also capable of. Therefore, it is suggested to use structurally defined HSs to investigate protein interactions and elucidate the impact of the interaction on sepsis (Axelsson et al., 2012). A research group used synthetic dodecasaccharides with or without 6-O-sulfation at the reducing end to reveal the importance of 6-O-sulfation on IL-8 inhibition by HSs (Jayson et al., 2015). Understanding HS structure preference to its binding proteins, as the study described above, is essential to optimize HS-based therapeutics in sepsis.

Damage associated molecular patterns (DAMPs) are nuclear, cellular, mitochondrial, or extracellular molecules passively released from damaged cells or actively released from immune cells in response to injury (Roh and Sohn, 2018; Gong et al., 2020). Several DAMPs, such as HMGB1, histones, extracellular cold-inducible RNA-binding protein (eCIRP), and heat shock proteins (HSPs), are recognized as critical inflammatory mediators that initiate and potentiate inflammation and can lead to organ dysfunction or death in sepsis (Denning et al., 2019; Gong et al., 2020). Similar to the electrostatic interactions between DNA and histones in the nucleus, these positively charged DAMPs are also reported to bind to HSs in sepsis.

Interestingly, the binding of DAMPs to HSs does not depend on electrostatic interactions alone. Recently, heparin was shown to trigger the formation of polymer complexes comprised of extracellular histones and cell-free DNA (cfDNA) in flowing blood. The polymer can be disrupted by DNase I and heparinase I/III, suggesting that heparin may form a tertiary complex with both negatively charged cfDNA and positively charged histone, although the mechanism is unknown (de Vries et al., 2019). In addition, the binding of heparin to HMGB1 is also shown to be independent of pH; other negatively charged molecules, such as sialic acid, do not bind to HMGB1 (Siddiqui et al., 2020). Their results suggest that factors other than electrostatic interaction contribute to HS/HMGB1 binding. A recent study from our group also indicated that the interaction between HMGB1 and extracellular histone 3 (H3) is HS size and sulfation pattern dependent in sepsis by applying a synthetic HS oligosaccharide library (Liao et al., 2023).

### HMGB1 as a DAMP in sepsis

HMGB1 (Amphoterin, HMG-1) is a highly conserved chromatin-binding protein that regulates transcription factors and stabilizes nucleosomes in normal physiology (Wang et al., 2006; Andersson et al., 2018). HMGB1’s extracellular inflammatory activity in sepsis was first identified in 1999 by administering the bacterial endotoxin (lipopolysaccharide, LPS) *in vitro* and *in vivo* models (Wang et al., 1999). The elevation of plasma HMGB1 is delayed but prolonged in septic mice, which can be observed from 8 to 32 h after LPS (i.p.) injection and from 18 to 72 h after cecal ligation and puncture (CLP) induced sepsis (Wang et al., 1999; Yang et al., 2004). In septic patients, the serum HMGB1 was significantly higher than in healthy participants and can persist up to 10 days after sepsis diagnosis (Karlsson et al., 2008; Karakike et al., 2019). The HMGB1 kinetics suggest that it is a promising target for sepsis as it may offer a broad therapeutic window to treat established septic patients compared to other acute inflammatory cytokines or chemokines.

**FIGURE 2:**
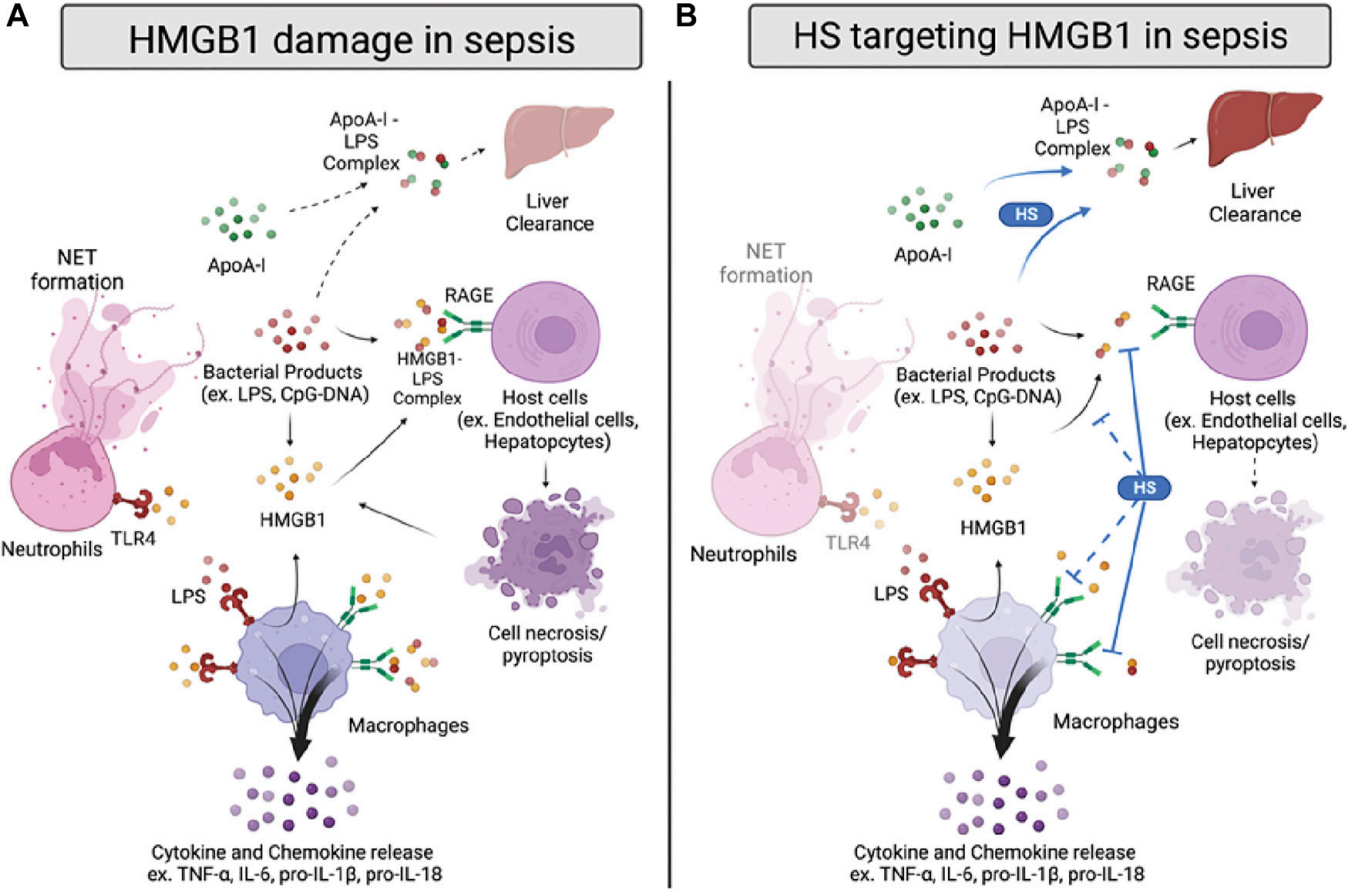
HMGB1 damage and proposed anti-HMGB1 mechanism of HSs in sepsis. **(A)** Infection sources, such as bacterial products, induce HMGB1 to be released from immune cells or damaged cells. HMGB1 binds to toll-like receptor 4 (TLR4) and receptor for advanced glycation end products (RAGE) on neutrophils and macrophages, inducing neutrophil extracellular trap (NET) formation and cytokines/chemokines release. HMGB1 also forms a complex with lipopolysaccharide (LPS), binding to RAGE on host cells and inducing cell pyroptosis. In addition, LPS forms a complex with apolipoprotein A-I (apoA-I), which can transport to the liver for clearance. **(B)** HSs reduce HMGB1-induced inflammation by inhibiting HMGB1/LPS complex binding to RAGE. Also, HSs facilitate apoA-I/LPS complex formation, replacing the LPS/HMGB1 complex. HSs are also proposed to reduce HMGB1/LPS complex formation and inhibit HMGB1/RAGE signaling in sepsis, yet the proposed mechanisms need further validation.

The proinflammatory activity of HMGB1 in sepsis can be attributed directly to its binding to receptors such as the Receptor for Advanced Glycation End-products (RAGE) and Toll-like Receptor 4 (TLR4) (Figure 2A). Also, HMGB1 can indirectly induce inflammation by interacting with other molecules, such as cytokines and bacterial products (Kang et al., 2014). For example, the HMGB1-RAGE axis is reported to activate macrophages to produce pro-IL-1β and pro-IL-18, trigger neutrophils-mediated injury, and increase the permeability of endothelial cells, mainly through the p38/ERK signaling pathway (Fiuza et al., 2003; He et al., 2012; Huang et al., 2012; Huebener et al., 2019). TLR4, on the other hand, binds to HMGB1 to induce the releases of TNF-α and IL-6 and promote the neutrophil extracellular trap (NET) formation (Yang et al., 2010; Tadie et al., 2013; Zheng et al., 2016). In addition, HMGB1 can bind to bacterial products, such as CpG-DNA or LPS, to facilitate their delivery into cells and further augment inflammation (Ivanov et al., 2007; Qin et al., 2009). Recently, a new mechanism of LPS delivery through coupling with HMGB1 was demonstrated by Deng and colleagues (Deng et al., 2018). Compared to LPS or HMGB1 alone, the LPS/HMGB1 complex induces dramatically higher TNF-α released from macrophages and increases LPS delivered into the cytosol. This resulted in increased caspase-11-dependent pyroptosis through binding to RAGE.

### HMGB1 and HSs

Extracellular HMGB1 has been found to bind to heparin/HS in previous studies, but the biological significance of the interaction between HMGB1 and HSs was not revealed until very recently. Heparin contributed significantly to the discovery of HMGB1, as HMGB1 was first isolated from the rat brain using heparin-Sepharose in 1987 (Rauvala and Pihlaskari, 1987). A few years later, it was discovered that HMGB1 was a ligand for syndecans, and binding was attributed to the HS chains, not chondroitin sulfate (Tam et al.) chains (Salmivirta et al., 1992). This result was confirmed in a later study that used mutant Chinese hamster ovary (CHO) cells, which lack HSs but have elevated CSs/dermatan sulfate (DSs). These mutant CHO cells could not bind HMGB1 compared to WT CHO cells (Xu et al., 2011). Moreover, treating human microvascular endothelial cells (HMVEC-c) with heparin lyases or protamine (a neutralizing heparin therapeutic) almost fully abolished HMGB1 stimulation, indicated by a significant reduction of phosphorylated Erk1/2 and p38. These results indicate that endothelial HSs are necessary for HMGB1 signaling. The binding site of HSs was further investigated by a biotinylation mapping strategy and mutagenesis studies, confirming that Lys-87, Lys-88, Lys-96, Lys-97, and Lys-150 contribute the most to HS binding to HMGB1. Interestingly, the authors later found that the mutant HMGB1 that lacks HS binding ability is still able to induce phosphorylation of Erk1/2 and p38 *in vitro*. RAGE, the receptor of HMGB1, was found to bind to HSs even in the absence of HMGB1. The RAGE/HS complex also can promote the oligomerization of RAGE, which is involved in MAPK/NF-κB activation (Zong et al., 2010; Xu et al., 2013). In addition to endothelial HSs, heparin has also been studied for HMGB1-RAGE binding. A study showed that heparin changes HMGB1 conformation by reducing β-sheet while inducing α-helix, with binding affinity K_D_ 9.77 × 10^−8^ mol/L (Ling et al., 2011). Treating murine macrophage Raw264.7 with heparin significantly decreases HMGB1-induced inflammation, potentially by interrupting HMGB1-RAGE signaling. However, whether the event is also involved in endothelial HSs interaction with HMGB1-RAGE signaling remains to be determined, preferably with structured defined HSs instead of a heparin mixture.

**FIGURE 3:**
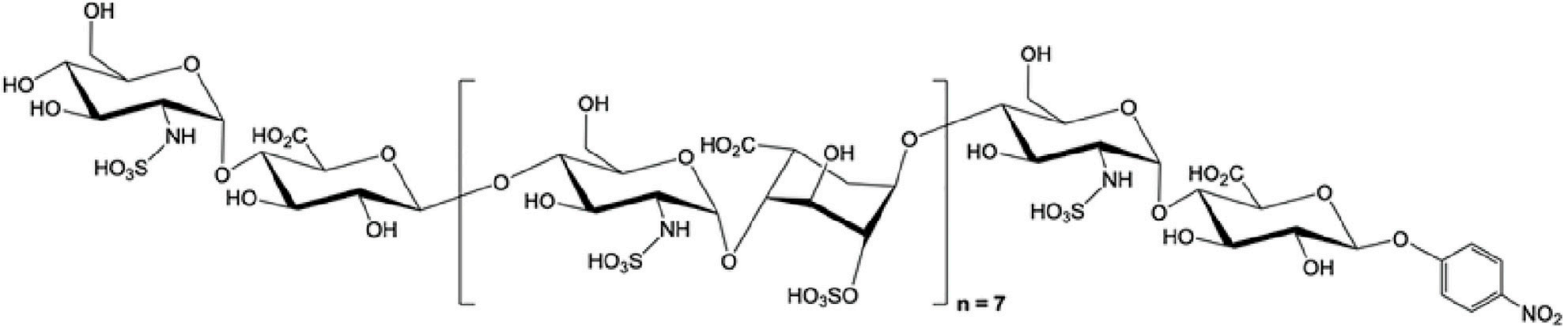
The structure of octadecassacharide (18-mer). The octadeacassacharide (18-mer) is produced by chemoenzymatic synthesis (Liao et al., 2023).

To further elucidate the binding between HMGB1 and HSs, our group utilizes chemoenzymatic synthesis to generate an HS library with various length and sulfation patterns (Liu and Linhardt, 2014). Previously, Xu and colleagues showed that HMGB1 binding to heparin heavily depends on N-sulfated HSs (Xu et al., 2011). In the acute liver injury models, including acetaminophen-induced drug toxicity and liver ischemia-reperfusion induced liver injury, we demonstrated that binding to HMGB1 has a specific HS length and sulfation level requirement (Arnold et al., 2020b; Arnold et al., 2020c). In the NS2S repeating disaccharide HS group, at least 18 saccharides (octadecasaccharide, Figure 3) are required to bind to HMGB1, with K_D_ at 168 nM (Arnold et al., 2020c). Reducing the HS length but maintaining the binding between HMGB1 and HSs is possible, but the sulfation degree needs to increase to NS2S6S (Arnold et al., 2020b). The binding between HMGB1 and HSs has shown therapeutic potential. Synthetic HSs reduced liver injury mediated by HMGB1 in both models of acute liver injury. The results set an example to further investigate HMGB1 and HSs relationship with structurally defined HSs and utilize this relationship for drug development.

Several studies have investigated HSs’ anti-HMGB1 activity in sepsis with various heparins and synthetic HSs (Figure 2B). Interestingly, most of the current evidence indicates that HSs’ beneficial binding effect on HMGB1 mainly targets the HMGB1/LPS—RAGE signaling pathway. For example, heparin treatment diminished the binding of HMGB1 to the cell surface under LPS stimulation in the murine macrophage Raw264.7 model (Li et al., 2015). Heparin also decreased the downstream p38/Erk1/2 pathway with a reduction of TNF-α induced by LPS/HMGB1 *in vitro*; however, it cannot affect inflammation induced by LPS alone. Also, Tang and colleagues indicated that heparin inhibits HMGB1/LPS binding. Inhibition leads to a reduction of LPS delivery to the cytosol, caspase-11 dependent pyroptosis, and sepsis-induced lethality (Tang et al., 2021). Moreover, the chemically modified non-anticoagulant heparin also could perform a similar effect as heparin, suggesting that the anti-inflammatory effect of heparin targeting HMGB1/LPS induced caspase-11 associated damage is independent of its anticoagulant activity.

To further elucidate the finding of HMGB1/LPS and HSs, our group used structurally defined HSs to determine their binding affinity between HMGB1 and investigated the reaction between HSs and HMGB1/LPS in sepsis. We found that HSs size dependently attenuated HMGB1/LPS induced inflammation (Liao et al., 2023). Interestingly, binding to HMGB1 by synthetic HS octadecassacharide (18-mer) is insufficient to dissociate the HMGB1/LPS complex. While the 18-mer binds to HMGB1, it cannot block HMGB1 binding to LPS in the endotoxemia model. Instead, HSs enlist the action of apolipoprotein A-I (ApoA-I), the main protein in high-density lipoprotein (HDL), to displace HMGB1 binding to LPS (Figure 2B). By increasing the non-toxic ApoA-I/LPS complex, the HS 18-mer reduces LPS/HMGB1-induced inflammation in sepsis. This protective mechanism of 18-mer was demonstrated in the LPS-induced endotoxemia and CLP mouse model. The mechanism involved in lipid modulation by HSs might contribute to the protective effect seen in septic mice co-administered atorvastatin and low molecular weight heparin (LMWH) (Jing et al., 2016). In this study, combined therapy of atorvastatin and LMWH significantly reduced lung injury, HMGB1, TNF-α, and IL-1β in CLP mice and improved survival rate. The potential therapeutic effect may be due to inducing HDL/apoA-I by atorvastatin and thus further augmenting heparin-facilitated apoA-I/LPS complex formation. Finally, in addition to findings of HSs on HMGB1/LPS complex, it is worth noting that the effect of HSs on HMGB1-RAGE induced damage cannot be excluded, as most of the synergetic effect of HMGB1/LPS complex was tested in a relatively low dose of HMGB1 and LPS.

### Histones as a DAMP in sepsis

Histones are highly conserved cationic nuclear proteins responsible for regulating the structure, stability, and availability of chromatin and DNA (Silk et al., 2017). Histones can be categorized into two subgroups; the core histones (H2A, H2B, H3, and H4) and the linker histones (H1 and H5) (Chen et al., 2014). The core histones have similar structures, binding to each other to form octameric cores and wrapping around DNA as nucleosomes, which are further connected by the linker histones as chromatins. The functional role of nuclear histones involves the regulation of epigenetic modifications and the replication of DNA.

During sepsis, histones are released as DAMPs from necrotic and apoptotic cells or activated immune cells, especially neutrophils, as part of neutrophil extracellular traps (NETs) (Chen et al., 2014). NETs are extracellular meshes composed of extracellular histones (H1, H2A, H2B, H3, and H4) and other proteins, including neutrophil elastase (NE), myeloperoxidase (MPO), and cathepsin G (Chapman et al., 2019). Specifically, citrullinated H3, which is generated by converting arginine to citrulline at residues 2, 8, and 17 by peptidyl-arginine deiminase 4 (PAD 4), has been used as a critical marker of NETs and proposed as a potential marker in sepsis (Li Y. et al., 2014; Thålin et al., 2020).

**FIGURE 4:**
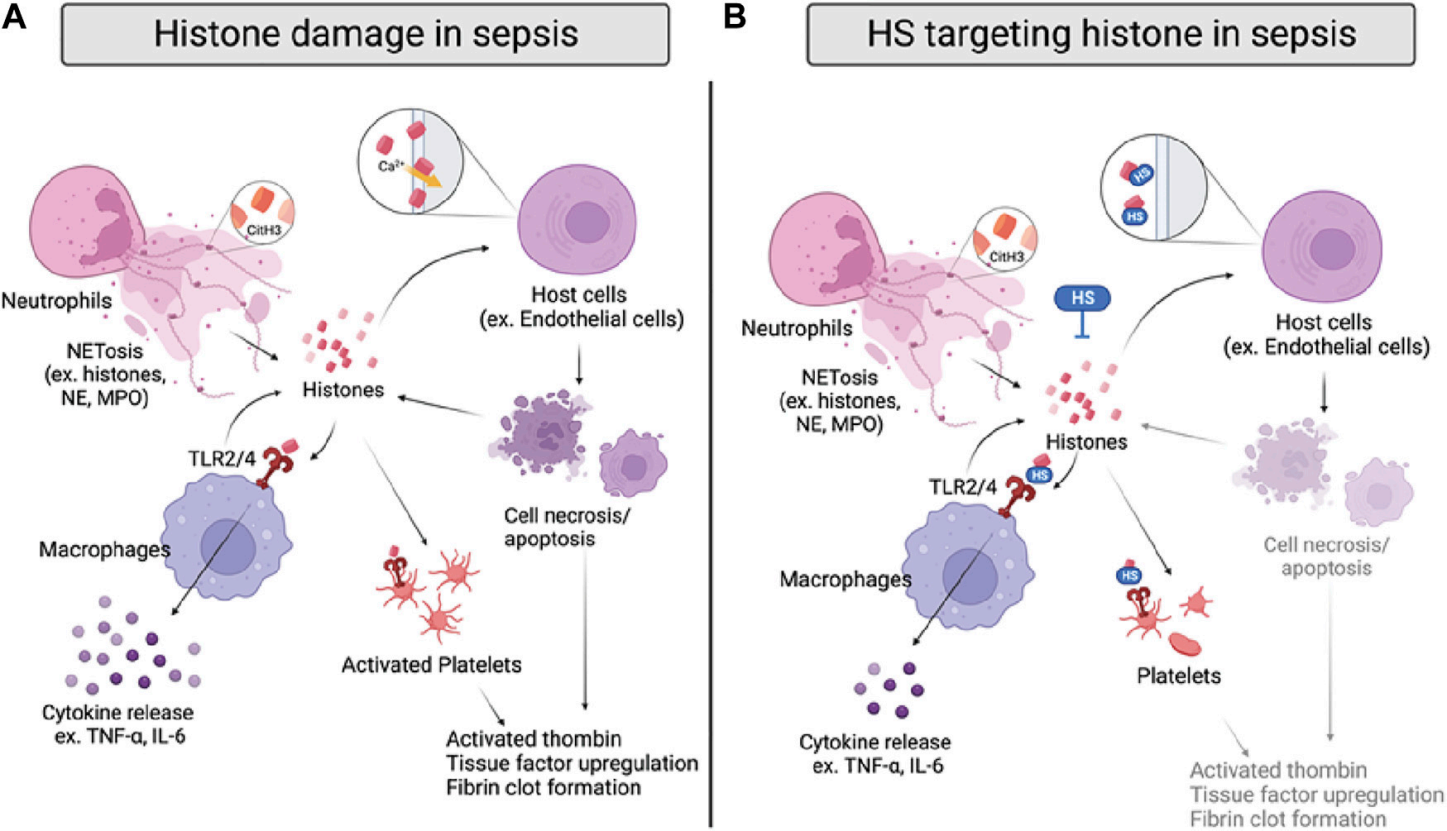
Histone damage and proposed anti-histone mechanism of HSs in sepsis. **(A)** Histones are released from activated neutrophils, macrophages, and damaged host cells during sepsis. Specifically, citrullinated histone 3 (CitH3) can be used as a NET marker. Extracellular histones directly interrupt cell integrity by interacting with cell membranes and inducing calcium influx, causing cell death. Histones also bind to TLR2/4 on macrophages and platelets, inducing cytokine release or coagulation. **(B)** HSs reduce histone-induced inflammation and coagulation by directly neutralizing histones, inhibiting their interactions with cell membranes and TLR2/4 on macrophages and platelets.

The detrimental effects of extracellular histones have been identified as crucial factors in sepsis, which can be attributed to direct interference of cell membrane integrity, inflammation, and coagulation (Figure 4A) (Xu et al., 2009; Li et al., 2021). First, extracellular histones exhibit direct cytotoxicity by binding to phospholipid-phosphodiester bonds of the endothelial and epithelial cell membrane, altering membrane permeability, and increasing calcium ion influx following cell death (Abrams et al., 2013; Xu et al., 2014). The disruption of calcium concentration by histones was also reported to cause cardiomyocyte damage in the CLP sepsis model (Kalbitz et al., 2015). Second, TLR2, TLR4, and TLR9 are implicated in histone-mediated damage and inflammation by activating NF-κB signaling pathways. Notably, activation of TLR2 and TLR4 by histones significantly increases cytokine TNF-α and IL-6; the mechanism was shown to contribute to septic kidney inflammation (Semeraro et al., 2011; Allam et al., 2012; Silk et al., 2017). Lastly, extracellular histones possess procoagulant activity, including promoting thrombin generation and activation, platelets activation, tissue factor upregulation, and fibrin clot formation (Ammollo et al., 2011; Simmons and Pittet, 2015; McDonald et al., 2017; Li et al., 2021). Of note, DAMP mechanisms of histones described above are intertwined; direct endothelial cell damage by histones also contributes to histone-mediated thrombosis, and impaired coagulation may further augment immune cell infiltration and proinflammatory chemokine/cytokines release.

As there are five subtypes of histones, it is important to note that the DAMP activity varies from H1 to H5. Specifically, H3 and H4 have been shown as the most proinflammatory and procoagulant extracellular histones (Xu et al., 2009; Ammollo et al., 2011). In human endothelial cells, H3 and H4 were indicated to be the most cytotoxic histones that significantly reduce cell viability. Also, H3 and H4 are the most potent histones to induce thrombin generation in platelet-poor plasma. Thus, developing anti-histone therapeutics may be more effective if focusing on targeting H3 and H4.

### Histones and HSs

Since histones are basic proteins enriched with lysine and arginine at the N terminal tail, it is rational to use negatively charged heparin to neutralize circulating histones and attenuate the overwhelming inflammation response in sepsis (Li et al., 2021). The notion has been tested in numerous studies with unfractionated heparin (UFH) and low molecular weight heparin (LMWH); both heparins effectively neutralize extracellular histones and improve survival rates of mice that underwent direct histones or H3 (i.v.) injection, LPS, and CLP-induced sepsis (Iba et al., 2015; Hogwood et al., 2020). The protective effect of UFH/LMWH could be attributed to reducing the inflammatory response, such as NF-κB/MAPK dependent secretion of TNF-α and IL-6, and dampening endothelial and epithelial cell damage to protect against sepsis-induced kidney and lung injury (Figure 4B) (Wang et al., 2015; Wang et al., 2020). In addition, UFH was also found to reduce histone-mediated coagulopathy, demonstrated by reduced mRNA expression of tissue factor, fibrinogen, thrombomodulin, and thrombosis in mice lungs (Hogwood et al., 2020).

Interestingly, Wildhagen and colleagues demonstrated that the anti-histone-induced cytotoxicity of heparin is independent of its anticoagulant activity (Wildhagen et al., 2014). Using non-anticoagulant heparin derived from UFH, the authors showed that the protective effect against H3-induced cell death and binding affinity to H3 *in vitro*. The overall protective effect of the non-anticoagulant heparin was further confirmed in mouse survival studies with the LPS and CLP sepsis models. In addition, the sulfation pattern and length requirement of HSs for their anti-histone effect have been investigated. In a recent study, HS oligosaccharides in different sulfation patterns and sizes were prepared by depolymerization and gel filtration from UFH (Zhang et al., 2017). The authors demonstrated that HS oligosaccharides must be at least ten sugar residues long and have at least N-sulfo-glucosamine in order to bind to the mixture of calf thymus histones. The requirement of N-sulfation is confirmed by another group using the whole blood cell model, showing that N-desulfated heparin lost its ability to attenuate histone-induced elevation of IL-6, IL-8, and tissue factor (Hogwood et al., 2020). Interestingly, tetrasaccharide and decasaccharide provide similar lung protective effects against a sub-lethal dose of histone injection in mice, but neither of them improves the survival rate of mice administered a lethal dose of histone (Zhang et al., 2017). However, both tetrasaccharide and decasaccharide protect against low and high doses of histone-induced cytotoxicity in the human lung microvascular endothelial cell (HPMVEC-ST1.6R) model. In our recent study, we apply chemoenzymatic synthesized HS oligosaccharides to determine binding affinity by SPR, anti-histone effect by human endothelial cell EAHy.926, and direct lethal dose of histone injection and CLP models (Liao et al., 2023). Similar to previous findings, we confirmed that binding to histone H3 is HS size and N-sulfation dependent, with 18-mer performing the best to protect against histone-induced cell death, inflammation, and lethality *in vitro* and *in vivo*. The different findings regarding the protective effects of HS oligosaccharides may be due to the HS length and purity. Further investigation on the structure-activity relationship between HSs and histone is recommended to optimize HS-based therapy targeting histones in sepsis.

## Potential therapeutics with HSs in sepsis

The characteristics of HSs have inspired therapeutic strategies in sepsis in three ways; first, developing HS-based treatments based on HSs’ interactions with numerous dysregulated proteins; second, preventing HS shedding with HSs derived or other small molecules; third, using HS fragments or HS related molecules as biomarkers in sepsis diagnosis. These three strategies have been tested in preclinical and clinical trials with promising results and are discussed below.

### HS-based potential treatments

#### UFH and LMWH

Recently, UFH and LMWH are also found to have potential protective effects in sepsis through interactions with critical proteins listed in Table 1, such as cytokines, chemokines, DAMPs, and adhesion molecules (Figures 4A, B). Interestingly, more studies attribute the protective effect to anti-inflammation rather than anticoagulation. Nevertheless, the anticoagulation effect of UFH is acknowledged in several studies, indicating that UFH reduces D-dimer, soluble fibrins, and thrombin-antithrombin (TAT) levels in plasma from LPS-challenged mice and healthy male volunteers (Pernerstorfer et al., 1999; Yang et al., 2019). Anti-inflammation effect of UFH/LMWH has been extensively investigated and revealed multiple pathways, such as neutralizing cytotoxic extracellular histones (Luan et al., 2014; Iba et al., 2015), inhibiting HMGB1/LPS mediated pyroptosis (Tang et al., 2021), interfering P-selectin/L-selectin associated neutrophil infiltration (Wang et al., 2002), and blocking inflammatory cascade by binding to IL-8, MCP-1, and HBP (Farrugia et al., 2018). However, the pro-infection effect of heparin was also reported in an *in vitro* study, showing that heparin facilitates LPS entry and activation of peripheral blood monocytes by binding to LPS-binding protein (LBP) and inducing IL-8 release (Heinzelmann and Bosshart, 2005). Such effect has also been observed with healthy volunteers given LPS and heparin, although in a small cohort (Heinzelmann and Bosshart, 2004). Nevertheless, UFH and LMWH are consistently shown to reduce organ injuries and improve survival in preclinical trials, suggesting an overall beneficial effect against sepsis. As inflammation and coagulation mechanisms are intertwined in sepsis, multi-functional heparin treatment such as UFH/LMWH is preferable as a drug candidate (Li and Ma, 2017; Hogwood et al., 2020). Further elucidation of the structure-function relationship of heparin in inflammation is required to advance HS-based therapy; investigations conducted with heparin derivatives and heparinoids are emerging.

The promising findings from preclinical studies with UFH/LMWH have inspired many clinical trials with septic patients, yet the results are conflicting (Table 2) (Li and Ma, 2017). Regarding large-scale randomized controlled trials, UFH/LMWH have been evaluated alone and in subgroup analysis combined with a once-approved sepsis treatment and two anticoagulants, including drotrecogin alfa (activated), tifacogin, and AT III in the 2010s (Warren et al., 2001; Abraham et al., 2003; Levi et al., 2007; Jaimes et al., 2009). Drotrecogin alfa (activated) is a recombinant activated protein C proposed to target extracellular histones in sepsis, and tifacogin is a recombinant tissue factor pathway inhibitor. The results show that UFH/LMWH is safe for septic patients except in combination with high-dose AT III therapy (Warren et al., 2001). The improvement of 28-day survival in severe sepsis was observed when giving UFH/LMWH in some trials, but the efficacy is not consistent. Further analysis by systemic reviews and meta-analysis with the above and other clinical trials have also been conducted, yet the conclusion is trending positive but is inconclusive (Wang C. et al., 2014; Zarychanski et al., 2015; Fan et al., 2016; Fu et al., 2022). Several clinical trials are still in progress, such as NCT04861922 (ClinicalTrial.org, retrieved on 12/1/2022).

**TABLE 2:** Heparin clinical trials.

Trial identifier	Year	Design	Indication	Testing treatment	Result
PROWESS	2001	• RCT^a^	• Severe sepsis	• DrotAA (24 μg/kg/h, 96 h)	• DrotAA significantly reduces mortality rate but may increase bleeding risk
		• 1,690 patients		• UFH ( ≤ 15,000 IU/day)	• DrotAA was approved in 2001, but withdrawn in 2011
KyberSept	2001	• RCT^a^	• Severe sepsis	• AT III (30,000 IU, 4d)	• ATIII cannot change mortality
		• 2,314 patients		• UFH/LMWH (s.c. ≤ 10,000 IU/day and/or flushes ≤ 2 IU/kg/h)	• Subgroup analysis: oCombine ATIII and heparin increase bleeding
					• Heparin may reduce mortality rate in placebo group
OPTIMIST	2003	• RCT^a^	• Severe sepsis with coagulopathy	• rTFPI (0.025 mg/kg/h, 4d)	• rTFPI cannot change mortality
		• 1,754 patients		• UFH/LMWH ( ≥ 1 dose before rTFPI)	• Subgroup analysis: o Heparin may reduce mortality rate in placebo group
NCT00049777 (XPRESS)	2007	• RCT	• Severe sepsis receiving DrotAA	• UFH (5,000 IU, bid)	• Prophylactic heparin for DrotAA treated patients may reduce mortality rate and incidence of ischemic stroke
		• 1,994 patients		• LMWH (40 mg, qd)	* DrotAA was withdraw from the market in 2011
NCT00100308 (HETRASE)	2009	• RCT	• Early diagnosed sepsis	• UFH (500 IU/h, 7d)	• UFH does not change length of stay, MOD, or mortality rate
		• 500 patients	• Bacterial infection		• UFH does not increase risk of bleeding
NCT04861922	Ongoing	• RCT	• Sepsis caused by abdominal infection	• UFH (10U/kg/h, 5d)	n/a
		• 100 patients			
NCT05208112 (HistoSeps)	Ongoing	• Single arm, open label	• Critically ill sepsis with organ dysfunction	• M6229 (non-anticoagulant heparin)	n/a
		• 16 patients			

^a^ Heparin teatment was not randomized.

^b^ The mortality rate is all-cause 28-day mortality rate unless specified.

*RCT, randomized controlled trial; DrotAA, drotregocin alfa (activated); ATIII, antithrombin III; rTFPI, recombinant tissue factor pathway inhibitor.

The conflicting results between heparin clinical trials in sepsis may be due to several reasons. First, sepsis is a complex disease with changing definitions. Sepsis was first defined in 1991 as a systemic inflammatory response to infection, modified in 2001, and updated to life-threatening organ dysfunction caused by a dysregulated response to infection in 2016 (van der Poll et al., 2017). The updated definition of sepsis removes the category of “severe sepsis,” defined as “sepsis and acute organ dysfunction.” Population and treatment decisions may differ before and after 2016, making the results before 2016 less applicable to the current situation (van der Poll et al., 2017). Particularly, the HETRASE trial that evaluated heparin efficacy in sepsis included participants who are not defined as septic patients now. New clinical trials are required to evaluate heparin’s efficacy in sepsis to date. Second, sepsis is a heterogenous symptom. Some studies suggested that UFH/LMWH only benefit severe septic patients with disseminated intravascular coagulation (Reitsma et al., 2007), while other analysis only included Asians showed more benefit with heparin treatment (Fan et al., 2016; Yamakawa et al., 2016). Identification of interested subgroups for heparin is recommended. Third, as alluded to above, the beneficial effect of heparin other than anticoagulant activity may not be fully explored (Li and Ma, 2017). During the COVID-19 pandemic, a retrospective study reported that LMWH significantly increases D-dimer and reduces IL-6 in COVID-19 patients, although in a small cohort (Shi et al., 2020). It is plausible that LMWH can also reduce the overwhelming inflammatory response in septic patients. Such anti-inflammatory effects may require a higher dose of UFH/LMWH, yet the bleeding risk limits the therapeutic window. Lastly, UFH/LMWH are HS mixtures; sulfation degree and patterns vary between sources. While 6-O-sulfation is reported as a critical sulfation pattern responsible for anti-inflammatory effect, UFH/LMWH from porcine mucosa is shown to have much higher 6-O-sulfated glucosamines than those from bovine mucosa (80% vs. 55%) (Hayashida et al., 2009; Mauri et al., 2019). Variations of anti-inflammatory effects between UFH/LMWH from different sources may further complicate the result.

#### UFH derivatives: Chemically modified heparins and non-anticoagulant heparins

**FIGURE 5:**
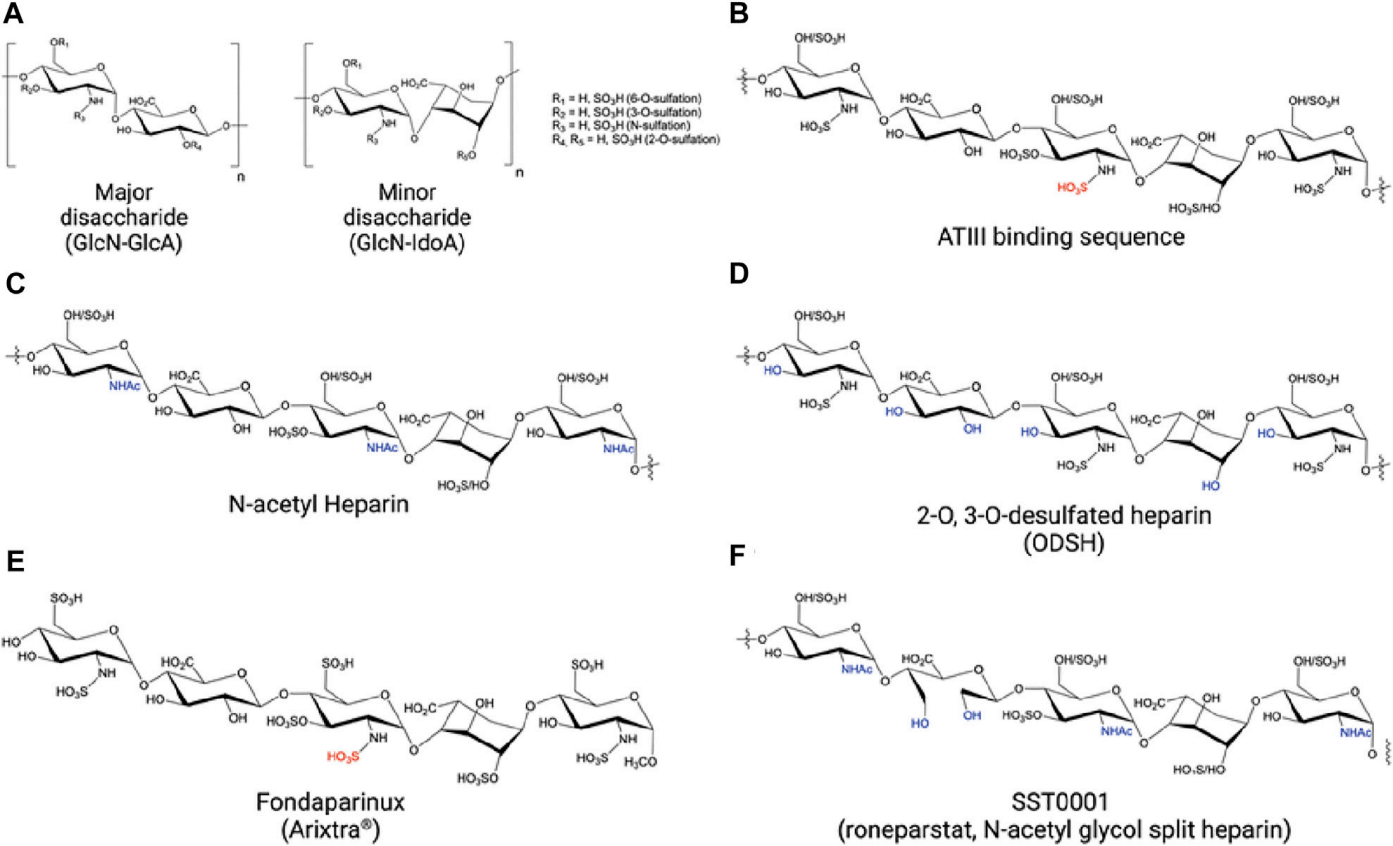
Structures of heparins. **(A)** Unfractionated heparin (UFH) and UFH derivatives, including low molecular weight heparin (LMWH), N-acetyl heparin, 2-O, 3-O-desulfated heparin (ODSH), and SST0001 are comprised of repeating disaccharide units. Glucosamine-glucuronic acid is more abundant that glucosamine-iduronic acid. Sulfation sites are varied as indicated. **(B)** The pentasaccharide binds to antithrombin III (AT III). The sequence is present in UFH and LMWH. **(C,D,F)** The structure of non-anticoagulant heparins. **(C)** The pentasaccharide in N-acetyl heparin. N-acetyl glucosamine eliminate anticoagulant activity. **(D)** The pentasaccharide in ODSH greatly reduces anticoagulant activity. **(E)** The structure of fondaparinux. **(F)** The pentasaccharide in SST0001 does not have anticoagulant activity.

Preclinical studies have shown that the protective effect of UFH/LMWH is independent of anticoagulation. Non-anticoagulant heparins can be generated by various chemical modifications with UFH and have been tested in preclinical models (Figures 5C–F). The non-anticoagulant UFH is now commercially available as N-acetyl heparin (Figure 5C) (Sigma, Iduron, etc.) (Nagasawa et al., 1977). N-acetyl heparin has been referred to as non-anticoagulant heparins (NAH) in many studies, shown to protect against sepsis-induced lethality and organ damage by targeting HMGB1/LPS complex (Tang et al., 2021), heparanase (Lygizos et al., 2013), and histones (Hogwood et al., 2020). In addition, a 2-O, 3-O-desulfated heparin (ODSH) with minimal anticoagulant activity has been generated and shown to increase activated protein C and inhibit histones in septic mice, with the safety confirmed in dog clinical trials for sepsis peritonitis (Figure 5D) (Kowalska et al., 2014; Mauro et al., 2022). Moreover, Wildhagen and colleagues generated an antithrombin affinity-depleted heparin (AADH) by using an antithrombin column to remove the anticoagulant activity of UFH (Wildhagen et al., 2014). AADH was shown with a similar protective effect against histone H3-induced cytotoxicity *in vitro* as UFH, significantly reducing neutrophil infiltration and lung injury and improving the survival of septic mice. A first-in-human clinical trial is currently recruiting critically ill septic patients for AADH treatment (NCT05208112, ClinicalTrial.gov, retrieved on 1/1/23).

#### Synthetic heparinoids

Synthetic heparin-like compounds are produced by chemical reactions and/or enzymatic synthesis. This allows researchers to have full control of HS length and sulfation pattern. Fondaparinux (Arixtra^®^) (Figure 5E), for example, is a chemically synthesized HS that only includes the essential pentasaccharide responsible for binding to ATIII (Samama and Gerotziafas, 2003). Compared to UFH/LMWH, fondaparinux is highly selective for anti-Xa activity vs. anti-IIa activity. Fondaparinux also has a longer half-life and higher bioavailability, although the synthesis is tedious, and an antidote is not available. In preclinical models, fondaparinux was shown to reduce *E. coli*-induced coagulopathy and inflammation in baboons (Keshari et al., 2020). In addition, fondaparinux mitigates white blood cell exhaustion and endothelial cell damage in LPS-challenged rats. However, it was less effective than LMWH (enoxaparin), suggesting that the size of fondaparinux is too short for the anti-inflammatory activity (Iba et al., 2012a). Non-anticoagulant fondaparinux was generated by oxidation of fondaparinux and shown to reduce inflammation in the kidney ischemia-reperfusion murine model but has not yet been tested in the sepsis model (Frank et al., 2006). Overall, fewer studies discuss the protective effect of fondaparinux in sepsis in preclinical and clinical settings. Based on current studies, fondaparinux’s anti-inflammatory activity seems less effective than UFH/LMWH. The difference is reasonable, as dysregulated protein targets such as HMGB1 require longer HSs and higher sulfation degree for binding and neutralization; fondaparinux is too short to bind to all inflammatory targets as UFH/LMWH (Liao et al., 2023).

Our group applies chemoenzymatic synthesis to produce structurally homogenous heparinoids. Using HS biosynthetic enzymes and sulfo donors in specific orders allows us to produce structurally defined HS oligosaccharides in various lengths and sulfation patterns. This method results in high purity and requires fewer synthetic steps than chemical synthesis (Liu and Linhardt, 2014). The chemoenzymatic synthesized HSs have high bioavailability and can also be selective to inflammation or coagulation targets with reversibility by protamine (Xu et al., 2017). More than a hundred synthetic HSs have been produced by chemoenzymatic synthesis, with or without anticoagulant activity, and used to elucidate the structure-function relationship between HSs and target proteins (Arnold et al., 2020a). In our previous studies, we used a synthetic HS library to investigate the binding requirement of HMGB1, showing that the anti-HMGB1 effect of HSs requires at least 18 saccharides or highly sulfated dodecassacharides (Arnold et al., 2020b; Arnold et al., 2020c). In a recent study, we demonstrate that a non-anticoagulant octadecasaccharide (18-mer) can be used as a multi-target therapy in LPS and CLP sepsis murine models by modulating pro-inflammatory extracellular H3, HMGB1, and anti-inflammatory ApoA-I (Figure 3) (Liao et al., 2023). Such structurally defined HS oligosaccharides may allow researchers to optimize carbohydrate-based therapy in sepsis by only choosing HS with the desired length and sulfation patterns. Proceeding to clinical trials for sepsis with synthetic heparin is recommended to further advance promising HS-based drug candidates.

### Potential treatments targeting HSs shedding

#### Heparanase inhibitors

Protecting the glycocalyx layer has been proposed as a potential anti-sepsis strategy (Iba and Levy, 2019; Uchimido et al., 2019). Heparins and their derivatives are shown as potential heparanase inhibitors (Vlodavsky et al., 2007; Ritchie et al., 2011). For example, SST0001 (Roneparstat), a non-anticoagulant heparin with N-acetylation and glycol split, has been tested in phase I clinical trial as heparanase inhibitors for multiple myeloma (Figure 5F) (Galli et al., 2018). Previously, we showed that heparanase could cleave the linkages between GlcA-GlcNS6S, GlcA-GlcNS3S, or GlcA2S-GlcNS, specifically reactive to GlcA-GlcNS6S in HSs (Peterson and Liu, 2010; Peterson and Liu, 2012). Thus, the HS sequences without the above disaccharides are probably resistant to heparanase and act as its competitive inhibitors.

Sulodexide, a highly purified portion from porcine intestinal mucosa, was reported as a heparanase inhibitor and is resistant to heparanase degradation. Sulodexide is comprised of HSs with iduronic acids and dermatan sulfate, which is not cleavable by heparanase. In a feces-induced peritonitis (FIP) model, sulodexide significantly improved survival in septic mice, facilitating glycocalyx restoration and reducing vascular permeability (Song et al., 2017). Our recent study using a largely heparanase-resistant synthetic octadecasaccharide also showed a renoprotective and anti-inflammatory effect (Liao et al., 2023). While the inhibition of heparanase by this octadecasaccharide is expected, further investigations are required to determine the interaction with heparanase *in vivo*.

Protein-based heparanase inhibitors have also been developed recently. A synthetic peptide 19-2.5, a peptide that was originally designed for anti-LPS treatment, was shown to reduce heparanase amount and activity in multiple organs, resulting in lessening circulating HS fragments in septic mice (Martin et al., 2015a). Heparanase-2 (HPSE2), an isoform of heparanase, is also identified as an endogenous heparanase inhibitor. In CLP-induced septic mice, HPSE2 was reported to reduce in plasma and kidney medullary capillaries. The reduction may indicate the loss of host regulation heparanase, as HPSE2 reduces HS shedding and LPS-induced TLR-4 signaling associated with heparanase activity (Kiyan et al., 2019). In addition, other drug modalities, such as neutralizing antibodies and siRNA, have also been proposed, further diversifying the choice of heparanase inhibitors (Vlodavsky et al., 2007). As each has different pharmacokinetics and metabolism pathways, multiple heparanase inhibitors available in clinics are ideal for clinicians to provide individualized treatment for septic patients. Although there are no approved heparanase inhibitors yet, the promising preclinical results from these drug candidates are expected to proceed further.

### Potential diagnostic tools related to HSs

#### Circulating HS fragments as sepsis biomarkers

Significantly increased HS fragments in serum have been repeatedly observed in septic patients, which indicates glycocalyx degradation and thus is proposed as a potential diagnostic marker (Martin et al., 2015b; Hippensteel et al., 2019; Uchimido et al., 2019). The elevation of circulating HS fragments can be detected as soon as the diagnosis of sepsis (at day 0) and last to at least 7 days, although the temporal change along sepsis recovery or progression remains to be determined. Whether these detected HS fragments are beneficial or detrimental is controversial. HS shedding is likely a balancing act; an inductive host defense mechanism in certain circumstances but harmful if out of control. Specifically, overwhelming circulating HS fragments may stimulate inflammation when treating healthy cells *in vitro*. Yet, they can act as a neutralizer and exert a cell-protective effect in the presence of dysregulated HSBPs, such as histones or the LPS/HMGB1 complex. Appropriate and timely HS shedding in septic patients is potentially beneficial by neutralizing pro-inflammatory chemokines, cytokines, and DAMPs.

#### HBP as a sepsis biomarker

HBP draws clinical interest for its correlation to sepsis severity, with seven completed clinical trials regarding its role as a biomarker for predicting sepsis prognosis, facilitating early diagnosis, and validating severe sepsis since 2015 (ClinicalTrial.gov, retrieved on 1/1/23). HBP encompasses multiple biomarker-like characteristics, including rapid released by neutrophils in response to infection, high sensitive and specific to sepsis in the emergency departments, correlated to sepsis-induced lung and kidney dysfunction, and has been validated by various independent research groups in different countries (Fisher and Linder, 2017). Based on available results from clinical trials, HBP is significantly increased in septic patients, especially those with organ dysfunction (Kahn et al., 2019). However, HBP does not perform better than other proposed sepsis biomarkers, such as procalcitonin (PCT) and C-reactive protein (CRP) in the trial. In another study, HBP levels were dramatically elevated in septic patients but failed to be used as a single biomarker to predict 28-day sepsis survival (Katsaros et al., 2022). While HBP is promising as a diagnostic tool for early diagnosis of sepsis, further investigation is needed to elucidate its role and correlation to severe sepsis. Ideally, a biomarker corresponding to sepsis resolution and progression will greatly benefit disease management.

## Conclusion

HSBPs, such as HMGB1 and histones, have been recognized as crucial modulators for inflammation and coagulation in sepsis. UFH/LMWH have been shown to be effective against sepsis by neutralizing dysregulated HSBPs, yet the mechanism is not fully understood. The efficacy has not been optimized due to its heterogeneous structure. Synthetic HSs with specific length and sulfation patterns are available recently, used as probes to elucidate mechanisms of HSBPs in sepsis and drug candidates to protect against sepsis-induced inflammation. Further investigations with homogenous HSs are expected to provide valuable insights into HS-based therapy. This may allow researchers to develop HS therapeutics with high specificity to various HSBPs and allow clinicians to optimize combined treatments for individuals based on the mechanisms of each drug. Ideally, developing HS-related biomarkers, such as HBP and circulating HS fragments, will significantly facilitate sepsis management by monitoring HS changes in sepsis, identifying the most benefitted subgroup for HS therapy, and evaluating the efficacy of HS-based therapies. Such combinations of HS-based therapies and diagnosis tools are promising to allow us to combat untreatable sepsis.
